# Pulse Consumption and Health Outcomes: A Scoping Review

**DOI:** 10.3390/nu16101435

**Published:** 2024-05-09

**Authors:** Naisi Zhao, Keyi Jiao, Yu-Hsiang Chiu, Taylor C. Wallace

**Affiliations:** 1 Public Health and Community Medicine, School of Medicine, Tufts University, Boston, MA 02111, USA; naisi.zhao@tufts.edu (N.Z.); jiaokeyi@sjtu.edu.cn (K.J.); yu-hsiang.chiu@tufts.edu (Y.-H.C.); 2 Department of Clinical Nutrition, College of Health Science and Technology, Shanghai Jiao Tong University School of Medicine, Shanghai 200025, China; 3 Think Healthy Group, LLC, Washington, DC 20001, USA; 4 School of Medicine and Health Sciences, George Washington University, Washington, DC 20037, USA; 5 Friedman School of Nutrition Science and Policy, Tufts University, Boston, MA 02111, USA

**Keywords:** pulse, seeds, plant proteins, Fabaceae, edible grain

## Abstract

Pulses—comprising the dry, edible seeds of leguminous plants—have long been lauded for their culinary flexibility and substantial nutritional advantages. This scoping review aimed to map the evidence on how pulses contribute to overall human health. Four electronic databases were searched for clinical and observational studies in English. We identified 30 articles (3 cross-sectional studies, 1 federated meta-analysis, 8 prospective cohort studies, 1 before-and-after study, and 17 randomized controlled trials) that met our inclusion criteria. Predominant among the pulses studied were lentils, chickpeas, common bean varieties (e.g., pinto, black, navy, red, kidney), black-eyed peas, cowpeas, and split peas. Consumption modalities varied; most studies examined mixed pulses, while five isolated individual types. In intervention studies, pulses were incorporated into diets by allotting a fixed pulse serving on top of a regular diet or by substituting red meat with pulses, offering a comparative analysis of dietary effects. The health outcomes evaluated were multifaceted, ranging from lipid profiles to blood pressure, cardiovascular disease risk and mortality, type 2 diabetes and glycemic control, metabolic syndrome indicators, inflammatory markers, oxidative stress biomarkers, and hormonal profiles. The most frequently assessed study outcomes included changes in low-density lipoprotein cholesterol, high-density lipoprotein cholesterol, systolic blood pressure, diastolic blood pressure, fasting blood sugar, hemoglobin A1c, waist circumference, and C-reactive protein or high-sensitivity C-reactive protein. This review should serve as a call to action for the scientific community to build upon the existing evidence, enriching our understanding of the nutritional and health-promoting attributes of pulses.

## 1. Introduction

Pulses—comprising the dry, edible seeds of leguminous plants—have long been lauded for their culinary flexibility and substantial nutritional advantages [1]. Pulses encompass a wide variety of types, with dry beans, dry peas, lentils, and chickpeas being the most universally recognized and consumed worldwide [2]. Other legumes that are harvested while still green (e.g., soybeans and peanuts) or garden vegetable varieties (e.g., green peas and beans) are not considered pulses, per the Food and Agriculture Organization of the United Nations (FAO) [3,4]. As a dietary staple for centuries, pulses offer a wealth of protein (which varies between 17% and 30% of their dry weight) and serve as a rich source of minerals (zinc, iron, calcium, magnesium), and thus are vital in diets that prioritize plant-based sources of nutrients [5,6,7,8]. US adults who consume pulses at any level of intake have higher energy-adjusted intakes of dietary fiber, folate, and magnesium compared with non-consumers; those with intakes of approximately 0.5 cup equivalents per day additionally have higher intakes of potassium, zinc, and iron and lower intakes of fat [9]. As dietary guidelines increasingly endorse plant-based eating patterns [10], it becomes imperative to ascertain the direct impact of pulses in the prevention and management of chronic conditions. The 2020–2025 Dietary Guidelines for Americans recommend adults consume 1.5 cups of beans, peas, and lentils per week as part of the vegetable group; however, due to their high protein content, they can also count toward the protein foods group [11].

In recent years, the profile of pulses has risen not only because of their nutrient density but also due to their contribution to health maintenance and ecological sustainability [10,12]. Pulses are increasingly acknowledged as a source of soluble and insoluble fiber, with a lower energy density and glycemic index compared with other carbohydrate-rich foods [13,14]. This slow digestibility makes them a favorable choice for the management of chronic diseases such as diabetes and cardiovascular conditions [15]. Moreover, their low-fat content and presence of healthy mono- and polyunsaturated fats, along with a spectrum of essential micronutrients and bioactive compounds with antioxidant properties, further establish pulses as a nutritional powerhouse [14].

Beyond nutrition, the environmental advantages of cultivating pulses are equally compelling [16]. Pulse crops act as natural carbon sinks and establish a symbiotic relationship with nitrogen-fixing bacteria, thus contributing to the reduction in greenhouse gas emissions and the enrichment of soil fertility [17,18,19]. Notwithstanding their known benefits, the intake of pulses varies considerably across different cultures and dietary guidelines, and there is still ambiguity regarding the optimal quantity for consumption that confers the best health outcomes [12]. 

This review aims to synthesize the current scientific evidence to elucidate the relationships between whole pulse consumption and health outcomes related to chronic disease prevention and management. Given the breadth and diversity of evidence, we have chosen a scoping review methodology that allows for a broader examination of the field, embracing diverse study designs and methodologies. This scoping review aims to provide a holistic view of the topic, pinpoint gaps in the research landscape, and chart areas for future systematic exploration. This endeavor will clarify the role of pulses in chronic disease prevention and management, thereby supporting the shift toward healthier, plant-based dietary patterns and emphasizing the need for their continued presence in both traditional and westernized diets.

## 2. Materials and Methods

The Preferred Reporting Items for Systematic Reviews and Meta-Analyses (PRISMA) Extension for Scoping Reviews (PRISMA-ScR) checklist was used to report the results of this study [20]. This scoping review followed the framework established by Arksey and O’Malley, consisting of the following: defining the review question, identifying relevant studies, charting the data, and summarizing the findings [21,22]. We adopted the Population–Concept–Outcome (P-C-O) approach to structure our search strategy and eligibility criteria (Table 1), and we centered our review around this question: “What is the extent of evidence supporting the recommendation of pulse consumption (Concept) in improving adult (Population) health outcomes (Outcome)”?

It was critical that the included studies allowed for the isolation of whole pulse consumption, either as an exposure of interest or as an intervention (e.g., a legume-enriched Dietary Approaches to Stop Hypertension [DASH] diet compared to a standard DASH diet). Eligible studies covered a range of pulses integral to diverse diets, including dry peas, chickpeas, lentils, and various beans such as pinto, black, kidney, navy, cow, mung, and fava. This definition adopted by FAO was employed to ensure that the selected studies were reflective of habitual dietary patterns and their sustained effects on health outcomes. In addition to the inclusion criteria outlined in Table 1, studies were excluded if their definition of pulse intake involved fresh garden bean varieties (e.g., green beans and green peas), oilseed legumes (e.g., soybeans and peanuts), isolated components of pulses (e.g., fiber and isoflavones), product forms of pulses (e.g., lupin-based beverages, fermented beans, lentil bread, lupin-enriched cereal, adzuki bean-based convenience food), or nutrients that may be derived from pulses but are also found in other foods (e.g., vitamin E, fiber, phytochemicals, etc.). 

**Figure 1:**
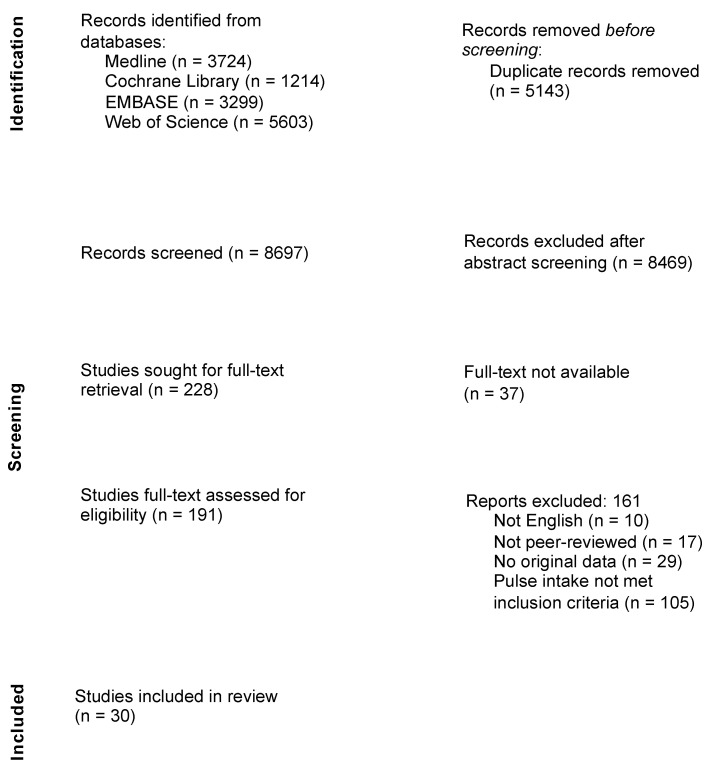
Literature search and study selection processes (PRISMA flowchart).

A trained librarian (P.S.) assisted in developing and conducting a comprehensive web-based search in the MEDLINE (PubMed) (National Library of Medicine; Bethesda, MD, USA), Web of Science (Clarivate Analytics; London, UK), Embase (Elsevier; Maryland Heights, MO, USA), and Cochrane (Wiley; West Sussex, UK) databases from inception to 21 July 2023. The search was designed to capture studies containing the concepts of pulse intake and health outcomes using relevant subject headings and text words adapted for the syntax of each database. The finalized search strategy is provided in File S1. No limits were placed on the results. Search results were downloaded and de-duplicated, and 8697 records were imported into Rayyan software (Rayyan Systems, Inc.; Cambridge, MA, USA). Independent screening was conducted by two teams of four reviewers. Starting with an initial screen of titles and abstracts, we applied our eligibility criteria, which resulted in the exclusion of 8469 records. The remaining records underwent subsequent dual full-text screening. Throughout the screening process, reviewers held regular meetings with a fifth reviewer to discuss and reconcile any discrepancies. The references to all included studies were hand-searched to ensure the retainment of all relevant articles. In total, 30 articles proceeded to the dual data extraction phase. The PRISMA flow diagram (Figure 1) visually maps the study selection process, including the reasons for exclusion. Finally, we reviewed the full text of the 30 identified articles and dually extracted data.

## 3. Results

### 3.1. Characteristics of Included Studies

The comprehensive search strategy identified 8697 records after the removal of duplicates. A total of 30 peer-reviewed articles met the inclusion and exclusion criteria (Table 2). These studies encompassed a diverse spectrum of study designs: 3 cross-sectional studies, 1 federated meta-analysis, 8 prospective cohort studies, 1 before-and-after study, 11 randomized crossover studies, and 6 randomized parallel trials. Geographically, the studies were distributed as follows: 13 from Iran (many of which pertained to the Therapeutic Lifestyle Changes project), 5 from the United States, 4 from Canada, 2 from China, 2 from Spain, 2 from multiple regions, and 1 each from Mauritius and Ecuador.

### 3.2. Pulse Consumption

In the eligible studies, a diverse array of pulses was tested in their whole form (raw, cooked, canned, or sprouted). The included articles were required to be specific about the pulse varieties they studied instead of only using general terms like *legumes* or *pulses*. Table 3 presents a matrix of pulses reported in the included studies. Predominant among the pulses studied were lentils (*Lens culinaris*), chickpeas (*Cicer arietinum*), common bean varieties (e.g., pinto, black, navy, and red kidney; *Phaseolus vulgaris*), black-eyed peas (*Vigna unguiculata*), cowpeas (*V. unguiculata*), and split peas (*Pisum sativum*). The consumption modalities varied; most studies (*n* = 25) examined mixed pulses, and five studies isolated individual types. Notably, two cohort studies [27,33] independently reported on lentils, chickpeas, and dry beans, and two RCTs [51,52] focused solely on lentils or lentil sprouts. Additionally, one randomized crossover study [42] evaluated black-eyed peas and pinto beans separately. In intervention studies, pulse was incorporated into diets by allotting a fixed pulse serving on top of a regular diet or by substituting red meat with pulses, offering a comparative analysis of dietary effects.

### 3.3. Outcome Measures

The health outcomes evaluated were multifaceted, ranging from lipid profiles to blood pressure, cardiovascular disease (CVD) risk and mortality, type 2 diabetes mellitus (T2DM) and glycemic control, metabolic syndrome indicators, inflammatory markers, oxidative stress biomarkers, and hormonal profiles. As detailed in Table 4, which compiles a matrix of reported health outcomes, the most frequently assessed metrics included low-density lipoprotein cholesterol (LDL-C), high-density lipoprotein cholesterol (HDL-C), systolic blood pressure (SBP), diastolic blood pressure (DBP), fasting blood sugar (FBS), hemoglobin A1c (HbA1c), waist circumference (WC), and C-reactive protein (CRP) or high-sensitivity CRP (hs-CRP).

### 3.4. Observational Studies

This review identified a variety of observational studies that reported original data, including cross-sectional studies, federated meta-analyses, and prospective cohort studies. The included cross-sectional studies, encompassing a total of 10,493 adults from Iran, Ecuador, and the United States, provided valuable insights into the role of pulses in promoting cardiovascular health and mitigating risks associated with metabolic disorders. These studies, notably large-scale in their participant base, offer a broad perspective and a snapshot of associations between pulse intake and their health impacts. A cross-sectional study conducted on older Iranian men found a positive association between higher non-oilseed legume intake and improved lipid profiles, as evidenced by increased levels of HDL-C and decreased levels of LDL-C [23]. Among 1997 participants from the Ecuadorian arm of the PURE study, an inverse relationship was observed between legume consumption and the prevalence of metabolic syndrome and T2DM [24]. Utilizing data from 8229 adults in the NHANES 1999–2002 survey, another cross-sectional study indicated that diverse bean consumption was linked to lower body weight, body mass index (BMI), and WC and a reduced risk of obesity [25]. Interestingly, the study also highlighted that bean consumers had lower SBP despite higher sodium intake.

Our comprehensive review identified eight prospective cohort studies, with median follow-up ranging from 4.3 to 13 years. The participant populations predominantly comprised older adults, although several studies also included middle-aged individuals. These populations varied widely, encompassing groups with high cardiovascular risk, low-income demographics, and diverse nationalities, including Ecuadorian, Iranian, and Mauritian cohorts. These studies collectively emphasized the positive impact of pulses on key health outcomes, notably CVD, T2DM, and hypertension. 

Two cohort studies examined the risk of developing T2DM at various levels of pulse consumption. In a PREDIMED cohort study involving 3349 older adults at high CVD risk in Spain, researchers found that higher lentil intake (8.88 g/d vs. 3.77 g/d) was associated with a 33% lower risk of developing T2DM over 4.3 years of follow-up [27]. A borderline significant inverse association was observed with chickpea consumption, whereas no significant associations were found with dry beans. In a cohort study of 1421 men and women in Mauritius followed for a median of 6 years, high consumption of pulses among women (16.7 g/MJ vs. 3.93 g/MJ) was associated with a 48% lower risk of developing abnormal glucose metabolism and a smaller increase in BMI [34]. In an effort to combine original data from multiple cohorts, a federated meta-analysis included 729,998 participants from 27 prospective cohorts from diverse regions around the world [26]. Pulse intakes were estimated as grams per day in each individual cohort. This analysis did not find an association between total pulse intake and T2DM overall or in different world regions. 

Other cohort studies focused on CVD risk and mortality, cancer mortality, and the risk of hypertension. A cohort of 41,243 Chinese adults was observed for a median of 8.9 years; this study reported a 29% lower risk of cancer mortality and a lower risk of major CVD events and all-cause mortality with 1–2 daily servings of non-soy legumes (150 g/serving) [29]. No further risk reduction was noted for more than 2 servings per week. In a large cohort study of 135,335 individuals spanning 18 countries across 7 regions, investigators found that higher non-soy legume intake was inversely associated with non-CVD death and total mortality [30]. The optimal consumption for maximum benefits was identified as 3 to 4 servings per day (150 g/serving). Over a median of 6.8 years of follow-up, consuming legumes more than three times per week was associated with a lower risk of CVD events among 5398 older Iranian adults compared with those with less frequent intake [31]. In a 13-year study involving 5432 Iranians, regular consumption of non-soybean legumes was linked to a lower risk of cardiovascular events. Long-term intake of legumes more than three times per week was associated with a 19% lower risk compared with individuals whose intake was less than once a week [32]. Over a median of 6 years of follow-up, higher consumption of total legumes (28.1 g/d vs. 11.75 g/d) and lentils (8.62 g/d vs. 3.88 g/d) was associated with a lower risk of cancer mortality in a Spanish cohort of 7212 older adults [33]. Specifically, those in the highest tertile of lentil consumption (8.62 g/d vs. 3.88 g/d) had a reduced risk of cancer mortality. This association was particularly strong in men, obese individuals, and those with diabetes. This study also observed similar correlations for CVD mortality in men, obese individuals, and those with diabetes. Lastly, in a study of 8758 Chinese adults with a median of 6.0 years of follow-up, consumption of total legumes, especially soybeans, was inversely associated with the risk of developing hypertension, particularly among those aged older than 65 years [28].

### 3.5. Intervention Studies

The included intervention studies (before-and-after studies, randomized crossover studies, and RCTs) consistently incorporated pulses into participants’ diets in controlled quantities, with the aim of evaluating the health outcomes of such dietary modifications against various control diets. Participant demographics were diverse, including general adult populations, individuals at higher risk of cardiovascular events, and specific groups such as patients with overweight and T2DM, polycystic ovary syndrome (PCOS), or abdominal obesity. The range of pulses used in these interventions was broad, encompassing lentils (green and red split), chickpeas, yellow split peas, and beans (pinto, fava, broad, black, and kidney). Among these studies, some assessed lipid profiles, blood pressure, and diabetes-related parameters, while others focused on inflammatory and oxidative stress biomarkers. The eligible intervention studies demonstrated a consistent pattern of health benefits associated with legume-enriched diets. 

Several studies have made specific adjustments to existing dietary patterns, such as the Therapeutic Lifestyle Changes (TLC) diet, by substituting servings of red meat with servings of non-soy legumes to create a legume-based version of these diets. In a series of crossover studies conducted in Iran, researchers evaluated the effects of a legume-based TLC diet on various health outcomes in patients with T2DM. Participants with diabetes adhering to a legume-based TLC diet exhibited significant reductions in oxidative stress indicators, with decreased malondialdehyde and oxidized LDL levels and increased nitric oxide and catalase activity [38]. The same legume-based TLC diet also led to a considerable rise in levels of serum adiponectin, a hormone involved in regulating glucose levels and fatty acid breakdown [39]. This increase was significant when compared to a legume-free TLC diet, although leptin levels remained unchanged across both diets, indicating a specific response of adiponectin to legume intake. Another randomized crossover study found that an 8-week intervention with a non-soybean legume-based TLC diet had beneficial effects on inflammatory markers closely linked with T2DM, including reductions in hs-CRP, IL-6, and TNF-α [44]. In addition, consuming the legume-enriched TLC diet led to notable improvements in fasting blood glucose, fasting insulin levels, and reductions in triglyceride concentrations and HDL-C in patients with overweight and T2DM [45]. Lastly, this review identified 1 TLC diet-related RCT [48] that reported women with PCOS experienced improved health-related quality of life scores with the legume-enriched TLC diet, with comparable reductions in body weight and insulin resistance to those consuming a TLC diet. The DASH diet is another example of substituting red meat with legumes. In a randomized crossover study, 300 Iranian men and women with T2DM experienced significant cardiovascular benefits when following a legume-based DASH diet [46]. This crossover study, which replaced a serving of bread and red meat with legumes for at least 5 days a week, resulted in a noteworthy reduction in SBP and urinary sodium levels, demonstrating the potential of legumes to enhance the traditional DASH diet for individuals managing T2DM.

Other interventions provided participants with pre-packed non-soy legumes to ensure standardized consumption levels and enrich the habitual diet with legumes to examine potential health benefits. In a before-and-after study, Canadian adults diagnosed with peripheral artery disease (PAD) consumed 0.25 to 0.5 cups of cooked non-soy legumes daily for 8 weeks and experienced reductions in serum cholesterol and ankle–brachial index; both measures are associated with CVD risk in patients with PAD [35]. Several randomized crossover studies provided participants with a fixed amount of cooked pulses daily. One Canadian crossover study demonstrated that adding cooked pulses to a regular diet reduced total cholesterol by 8.3% and LDL-C by 7.9% compared to the regular diet alone [36]. For participants with “borderline high” levels of cholesterol, the pulse-based diet significantly reduced total cholesterol by 6% more than the regular diet. Additionally, there was a small but significant decrease in body fat percentage among those with body fat above the normative value for their age. In the other US crossover study, male participants consumed a legume-enriched, low-glycemic index diet and experienced a significant increase in fasting glucose concentration [37]. Compared with a healthy US diet, this legume-enriched diet also led to significantly reduced fasting CRP and marginally reduced concentrations of soluble tumor necrosis factor receptor II. Two articles reported on a randomized crossover study that provided 260 g of cooked pinto beans and brown lentils to Iranian men and women who were first-degree relatives of patients with T2DM [40,41]. This legume-enriched habitual diet led to a significant reduction in hs-CRP levels over 6 weeks of intervention, especially in female participants [40]. However, no significant effects on anthropometric measures, blood pressure, glycemic indices, or lipid profiles were observed [41]. Two US randomized crossover studies also provided pinto beans as part of pulse enrichment [42,43]. One randomized crossover study showed that consuming 0.5 cups of canned pinto beans daily over 8 weeks led to significant reductions in total cholesterol and LDL-C in a mildly to moderately insulin-resistant US population [42]. The other study explored the effects of a healthy US diet enriched with a mixture of cooked beans on inflammatory markers [43]. This 4-week intervention resulted in decreased fasting plasma leptin concentrations in US men but had no effect on fasting ghrelin concentrations, regardless of their insulin resistance status. 

An RCT reported that a hypocaloric diet enriched with 1 cup of cooked non-soy legumes notably reduced WC and SBP in premenopausal women, with triglyceride levels decreasing significantly between 3 and 6 weeks [47]. Interestingly, although fasting insulin and homeostasis model of insulin resistance (HOMA-IR) levels initially increased in both study arms, they returned to baseline after 6 weeks in the legume-enriched group. In another study in which premenopausal women with abdominal obesity followed the same legume-enriched hypocaloric diet, a significant reduction in hs-CRP levels was observed [50]. This study also highlighted that the legume-enriched diet did not affect fasting concentrations of total cholesterol, whereas the diet without legumes led to an increase. In another RCT, overweight men and women received 5 cups of cooked non-soy legumes per week in addition to following an energy-restricted diet [49]. This RCT found reductions in WC and SBP in the pulse-enriched group without changes in body weight, BMI, or DBP. This study also noted improvements in HbA1c, HDL-C, and C-peptide levels, suggesting a positive impact of pulses on glycemic control and lipid profiles.

In the investigation of lentil intake and its effects on metabolic health, men and women with abdominal obesity were randomized into three groups of green lentil consumption, with one group having no lentils and another completely replacing meat with lentils [51]. The findings indicated an improvement in insulin resistance, as shown by a reduction in HOMA-IR. Another lentil-focused RCT provided overweight and obese patients with T2DM with 60 g of lentil sprouts daily over an 8-week period [52]. The study found that the addition of lentils was linked with a beneficial alteration in lipid profiles, including a significant reduction in triglycerides and oxidized LDL, and an increase in HDL-C was observed, underscoring the positive influence of lentil consumption. 

### 3.6. Identifying Research Gaps Related to Study Design

**Figure 2:**
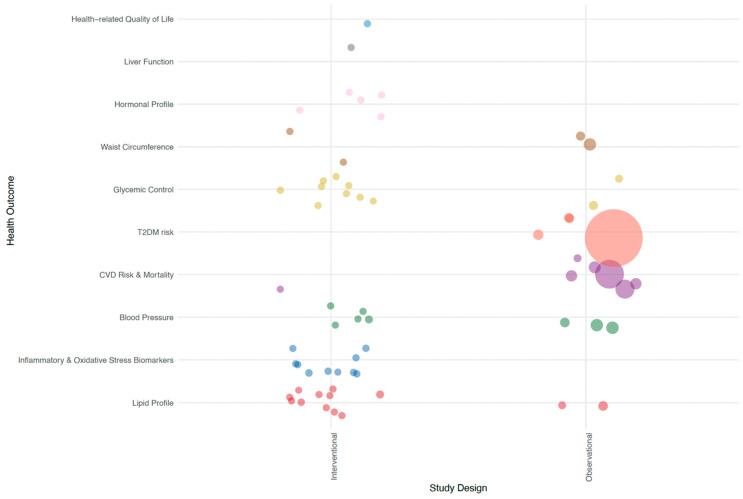
Bubble plot of health outcome categories by study design. Every bubble corresponds to an individual study, with the bubble’s dimension reflecting the number of participants in that study. Abbreviations: CVD, cardiovascular disease; T2DM, type 2 diabetes mellitus.

Figure 2 delineates the range of health outcomes investigated in the pulse intake studies in this review, distinguishing between interventional and observational research designs. Interventional studies predominantly focused on biomarker health outcomes, examining a variety of indicators such as lipid profiles, markers of inflammation and oxidative stress, blood pressure, glycemic control, and hormonal profiles. In contrast, observational studies were more inclined to investigate long-term chronic disease outcomes, particularly the risk and mortality associated with T2DM and CVD, conditions that manifest over extended periods. Consistent with research norms, the observational studies featured substantially larger participant cohorts compared with those in interventional studies.

## 4. Discussion

This scoping review synthesized evidence from 30 peer-reviewed articles, underscoring the role of pulse consumption in enhancing health outcomes, particularly for individuals with chronic conditions like T2DM and CVD. The studies reviewed here, with diverse designs and populations spanning several countries, consistently illustrated pulses as beneficial for health, aligning with dietary guidelines that advocate for plant-based diets. 

The body of research included in our review consistently underscores the integral role of pulses in the management of T2DM and the enhancement of glycemic control. Notably, diabetes emerges as the outcome with the largest sample size among the observational studies considered. Cohort studies, including PREDIMED, have observed a marked reduction in T2DM risk correlating with increased consumption of lentils. Similarly, evidence from Mauritius suggests a strong association between a high intake of pulses and a reduced risk of abnormal glucose metabolism, particularly in women. These findings, however, are nuanced by a comprehensive federated meta-analysis that challenges the universality of this correlation, pointing to regional variations in diet or pulse preparation methods as potential modifiers of this relationship.

Interventional studies, which have the largest sample sizes for examining lipid profiles, corroborate the beneficial effects of pulses on key cardiovascular biomarkers, such as LDL-C and HDL-C. These studies, encompassing diverse populations and dietary contexts, consistently document improvements in fasting glucose levels and insulin sensitivity when pulses are incorporated into diets. Noteworthy are the interventions where pulses either replaced red meat or were added as fixed servings; both scenarios demonstrated pulses’ efficacy in dietary quality enhancement and favorable health outcomes. There is likely sufficient clinical evidence to support a future systematic review and dose–response meta-analysis to investigate the effects of pulse intake on blood lipid levels. Other research areas are still emerging, with existing studies exhibiting heterogeneity.

Evidence suggests that pulses are valuable in dietary strategies for diabetes management and cardiovascular health. Nonetheless, the potential of pulses can only be fully realized through comprehensive research that disentangles their effects as a standalone dietary component and in concert with other foods. The influence of different cooking methods on the glycemic index of pulses, for example, remains an area ripe for investigation. Future studies that meticulously assess these aspects will be instrumental in refining dietary recommendations for individuals with diabetes and optimizing the health benefits attributed to pulse consumption.

The included studies were conducted in a small number of countries, with a considerable number from Iran, which may reflect cultural dietary patterns and regional research priorities. The concentration of studies in Iran, many related to the TLC project, emphasizes the region’s recognition of the importance of dietary interventions in chronic disease management. However, it also indicates a need for broader research across diverse populations to generalize these findings globally. Moving forward, systematic reviews and meta-analyses are needed to consolidate these findings and quantify the effects of pulses on health outcomes accurately. Longitudinal studies could further elucidate the long-term impacts of pulse consumption. Additionally, research exploring the environmental benefits of pulses alongside their health impacts could provide a more holistic view of their role in sustainable food systems.

This scoping review, conducted with a robust methodology anchored by Arksey and O’Malley’s framework and adhering to PRISMA-ScR guidelines, represents a methodological strength, ensuring thoroughness and transparency. The use of the P-C-O strategy facilitated a focused yet expansive capture of relevant studies, while the lack of publication date restrictions allowed for a comprehensive temporal scope. However, the review’s limitations include variability in pulse consumption modalities across studies, potentially affecting outcome measure consistency, and a notable gap in the literature regarding the underlying mechanisms of pulse impact on health, which is crucial for substantiating dietary guidance. Additionally, the restriction on English-language articles may introduce a linguistic bias, potentially overlooking significant global research findings. The absence of an assessment of evidence strength based on study design could also result in an incomplete appraisal of the quality of the evidence. To address these limitations, future research should extend beyond English-language literature and employ a critical evaluation of the evidence, considering study design, to enhance the review’s breadth and depth. Despite these limitations, the review findings affirm the health benefits of pulses and highlight the need for further investigation to fully elucidate their role in health outcomes.

## 5. Conclusions

In summary, this scoping review maps existing research and narratively highlights the potential role of pulses in maintaining health and preventing chronic disease. The greatest amount of clinical evidence highlighted within this scoping review pertains to the effect of pulses on blood lipid levels; this area could warrant future investigation through systematic review and dose–response meta-analysis. It is apparent that future research should more precisely and uniformly report pulse type(s) and intake quantities, so systematic reviews and dose–response meta-analyses can accurately pinpoint optimal intake levels needed to achieve desired effects on health. Ultimately, the review serves as a call to action for the scientific community to build upon existing evidence gaps to enrich both our understanding of the nutritional and health-promoting attributes of pulses and to strengthen current public health recommendations.

## Figures and Tables

**Table 1:** Structure of review question and inclusion criteria.

Characteristic	Inclusion Criteria
Population	Human adult participants (age ≥ 18 y), except pregnant women
Concept/intervention	Must involve the consumption of whole pulses—whether raw, cooked, canned, or sprouted—for a period extending beyond 2 wk Must quantify pulse consumption, specifying the amount in servings per day or week, or grams per day or week, for at least 2 distinct categories
Outcomes	Any direct measure of physical health that could be influenced by pulse consumption. This definition of included outcomes was operationalized as any measurable endpoints that could be categorized as lipid profile, blood pressure, inflammatory biomarkers, oxidative stress biomarkers, glycemic control, metabolic syndrome and waist circumference, liver function, hormonal profile, CVD risk and mortality, T2DM risk and mortality, or overall function and well-being. This encompassing criterion allowed us to consider a comprehensive range of indicators reflective of the multifaceted impact of pulse intake on health.
Study design	Analytical studies (exclude case series, case reports, and qualitative studies)
Other	Research studies presenting original data published in peer-reviewed journals. Available in full text in the English language.

**Table 2:** Characteristics of studies investigating the effect of pulse consumption on health outcomes.

Reference	Study Design	Country	Sample Size and Population	Pulse Type	Dietary Assessment	Pulse Dose	Duration	Main Outcomes	Main Findings
Askari et al., 2021 [23]	Cross-sectional	Iran	267 men (age ≥ 60 y)	Lentils, chickpeas, cotyledon, beans, and peas	FFQ	Dose (times/wk): T1: ≤10.51; T2: >10.51 and ≤14.56; and T3: >14.56	NA	LDL-C HDL-C TAG FBS FRS	↓ LDL-C serum levels ↑ HDL-C serum levels No association with FRS, TAG serum level, and FBS
Baldeón et al., 2021 [24]	Cross-sectional	Ecuador	1997 participants in the Ecuadorian cohort of the international PURE epidemiological study (72.1% women; mean age 51 ± 10 y; 62.8% low or medium income)	Beans, lentils, peas, fava beans, chickpeas, and lupins	FFQ	1 serving = 150 g cooked legumes Dose (servings/d): low: ≤0.5; moderate: 0.51–1; and high: >1	NA	T2DM MetSyn WC Fasting TG SBP DBP Cardiometabolic risk factors (TC, LDL-C, HDL-C, and TG)	↓ Odds in the probability of present MetSyn and T2DM ↓ Odds in the probability of presenting hypertension ↓ SBP ↓ DBP No association with cardiometabolic risk factors
Papanikolaou et al., 2008 [25]	Cross-sectional	US	8229 men and women (age ≥ 20 y) from the 1999–2002 NHANES; excluded pregnant or lactating females	Baked beans, variety beans (pinto, kidney, etc.), variety beans and/or baked beans	24 h food recall	Baked beans (mean servings/d): 2.7 ± 0.2 vs. 3.3 ± 0.04 Variety beans (mean servings/d): 1.7 ± 0.08 vs. 0.14 ± 0.01 Variety beans and/or baked (mean servings/d): 1.5 ± 0.05 vs. 0.12 ± 0.01	NA	Body weight WC SBP DBP	↓ Body weight ↓ WC ↓ SBP
Pearce et al., 2021 [26]	Federated meta-analysis	Multiple regions	729,998 participants from 27 prospective cohorts	Pulse (defined as consumption of peas, beans, chickpeas, and lentils)	Of the 27 cohorts, 17 used semi-quantitative FFQs, 4 used a quantitative dietary questionnaire, 3 used an interviewer-administered dietary history, 2 used a 24 h recall, and 1 used either an FFQ or a quantitative dietary questionnaire	Pulse intakes were estimated in each individual cohort (in g/d), and incident rate ratios were calculated per 20 g/d higher intake of pulse	NA	Incident T2DM	No association with T2DM (overall or region-specific)
Becerra-Tomás et al., 2018 [27]	Prospective cohort	Spain	3349 men (age 55–80 y) and women (age 60–80 y) without CVD at enrollment but with high cardiovascular risk	Lentils, chickpeas, dry beans, and fresh peas	Semi-quantitative FFQ	Lentils (g/d): Q1: 3.77; Q2: 5.62; Q3: 7.66; and Q4: 8.88 Chickpeas (g/d): Q1: 3.05; Q2: 4.35; Q3: 6.15; and Q4: 8.59 Dry beans (g/d): Q1: 2.16; Q2: 4.16; Q3: 5.67; and Q4: 8.45	Follow-up: 4.3 y	Incident T2DM	Lentil: ↓ Risk of T2DM Chickpea: ↓ Risk of T2DM Dry bean: No association with T2DM
Guo et al., 2020 [28]	Prospective cohort	China	8758 men and women (not pregnant, age ≥ 30 y)	Non-soybean dry legume consumption (e.g., mung, adzuki, red kidney, and pinto)	24 h food recall Household food inventory weighing	Non-soybean beans (g/1000 cal): Q1: 6.3; Q2: 15.4, Q3: 27.3; and Q4: 50.3	Median follow-up: 6.0 y	Incident hypertension	Total legumes: ↓ Hypertension risk Non-soybean: No association with hypertension
Liu et al., 2021 [29]	Prospective cohort	China	41,243 men and women (age 35–70 y)	Beans, lentils, chickpeas, black beans, peas, and black-eyed peas	FFQ	1 serving = 150 g/d Dose per group (servings/d): Group 1: <2; Group 2: 2–3; Group 3: 3–4; Group 4: 4–5; and Group 5: ≥5	Median follow-up: 8.9 y	Incident CVD CVD mortality Incident cancer Cancer mortality All-cause mortality	↓ Cancer mortality ↓ Major CVD ↓ All-cause mortality No association with CVD mortality
Miller et al., 2017 [30]	Prospective cohort	18 countries in 7 regions (North America and Europe, South America, the Middle East, South Asia, China, Southeast Asia, and Africa)	135,335 men and women (age 35–70 y)	Beans, black beans, lentils, peas, chickpeas, and black-eyed peas	FFQ	1 serving = 150 g/d Dose per group (servings/d): Group 1: <1; Group 2: 1–2; Group 3: 2–3; Group 4: 3–4; Group 5: 4–5; Group 6: 5–6; Group 7: 6–7; Group8: 7–8; and Group 9: ≥8	Median follow-up: 7.4 y	CVD mortality Non-CVD mortality Total mortality Major CVD (including MI, stroke, etc)	↓ Non-CVD mortality ↓ Total mortality ↓ CVD mortality (in the minimally adjusted models)
Nouri et al., 2016 [31]	Prospective cohort	Iran	5398 men and women (age ≥ 35 y)	Legumes other than soybean	FFQ	Dose (times/wk): T1: 0–1; T2: 1–3; and T3: ≥3	Median follow-up: 6.8 y	Incident CVD events (CHD, including fatal and nonfatal MI, sudden cardiac death and unstable angina, or stroke)	Non-soybean: ↓ CVD events
Nouri et al., 2021 [32]	Prospective cohort	Iran	5432 men and women (age ≥ 19 y), mentally competent and not pregnant	Soybean and non-soybeans (lentils, peas, beans, and mung beans)	FFQ	Non-soybean legumes (times/wk): 1–3 vs. 0–1; ≥3 vs. 0–1 Overall legumes (times/wk): 1–3 vs. 0–1; >3 vs. 0–1	Median follow-up: 13 y	Incident CVD events (CHD, including fatal and nonfatal MI, sudden cardiac death and unstable angina, or stroke)	Long-term non-soybean intake: ↓ CVD events
Papandreou et al., 2019 [33]	Prospective cohort	Spain	7212 men (age 55–80 y) and women (age 60–80 y) without CVD at enrollment but with high CVD risk	Lentils (*Lens culinaris*), chickpeas (*Cicer arietinum*), dry beans (*Phaseolus vulgaris*) and fresh peas, (*Cajanus cajan*)	FFQ	Consumption reported as average daily intake in grams, adjusted for total energy intake: Total legumes (g/d): T1: 11.75; T2: 18.42; and T3: 28.10 Lentils (g/d): T1: 3.88; T2: 6.13; and T3: 8.62 Chickpeas (g/d): T1: 3.55; T2: 4.33; and T3: 8.39 Dry beans (g/d): T1: 3.24; T2: 4.21; and T3: 8.95	Median follow-up: 6 y	Cancer mortality CVD mortality	Total legumes: ↓ Cancer mortality in all ↓ CVD mortality in men ↓ Cancer mortality in men ↓ Cancer mortality in obese individuals ↓ Cancer mortality in T2DM Lentil intake: ↓ Cancer mortality in all Dry beans intake: ↑ CVD mortality
Wennberg et al., 2015 [34]	Prospective cohort	Mauritius	1421 men and women (age 30–64 y)	Pulse (e.g., lentils, chickpeas, beans, and peas)	FFQ 24 h food recall	Energy-adjusted intake of pulses (g/MJ) Men: T1: 3.7; T2: 7.96; and T3: 15.4 Women: T1: 3.93; T2: 8.34; and T3: 16.7	Median follow-up: 6 y	Abnormal glucose metabolism (defined as T2DM, impaired glucose tolerance, or impaired fasting glucose BMI WC	Women: ↓ Risk of developing abnormal glucose metabolism ↑ BMI Men: No association with the risk of abnormal glucose metabolism ↑ BMI ↑ WC
Zahradka et al., 2013 [35]	Before-and-after	Canada	26 men and women (age ≥ 40 y) with PAD	Beans (pinto, kidney, black, and navy), peas, lentils, chickpeas	3-day dietary record	Week 1: 0.25 cup cooked legumes/d Week 2–8: 0.5 cup cooked legumes/d	8 wk	TC LDL-C Fasting glucose level Ankle–brachial index	Non-soybean legume: ↓ Serum cholesterol ↓ Ankle–brachial index No significant change in LDL-C, TC
Abeysekara et al., 2012 [36]	Randomized crossover	Canada	87 men and women (mean age 59.7 y; mean BMI 27.5 ± 4.5)	Green lentils, red split lentils, chickpeas, yellow split peas, and pinto, fava, broad, black, and kidney beans	FFQ	Intervention: 2 servings/d or 150 g/d dry weight or 250 g/d wet weight cooked lentils, chickpeas, beans, and peas. Pulses included green lentils, red split lentils, chickpeas, yellow split peas, and pinto, fava, broad, black, and kidney beans Control: regular diet	8 wk	TC LDL-C TAG HDL-C CRP	↓ TC ↓ LDL-C No significant change in HDL-C, TAG, CRP, glucose, or insulin
Hartman et al., 2010 [37]	Randomized crossover	US	64 men (mean age 54.5 y) characterized for colorectal adenomas and IR status	Navy, pinto, kidney, and black beans	3-day dietary record	Intervention: legume-enriched, low-glycemic index diet that contained~250 g legumes/d (including navy, pinto, kidney, and black beans) Control: a healthy US diet	4 wk	CRP C-peptide levels sTNFRI sTNFRII Fasting glucose	↓ CRP ↑ Fasting glucose No significant change in C-peptide, sTNFRI, sTNFRII
Mirmiran et al., 2018 [38]	Randomized crossover	Iran	40 men and women (age 50–75 y, BMI 25–30) with diabetes	Non-soybean (e.g., lentils, chickpeas, peas, and beans)	3-day dietary record	Intervention: non-soy legume-based TLC diet, same as the legume-free TLC diet (diet follows the dietary recommendations of TLC guidelines), except 2 servings red meat were replaced with different types of legumes 3 d/wk Control: legume-free TLC diet, macronutrient composition of 50–60% carbohydrates, 15% protein, and 25–35% of energy from fat	8 wk	Oxidative stress biomarkers Serum MDA Serum ox-LDL Serum NO CAT activity	↓ MDA ↓ ox-LDL ↑ NO ↑ CAT activity
Mirmiran et al., 2019 [39]	Randomized crossover	Iran	31 men and women (age 50–75 y; BMI 25–30) with T2DM	Lentils, chickpeas, peas, or beans	3-day dietary record	Intervention: non-soy legume-based TLC diet, same as the legume-free TLC diet (diet follows the dietary recommendations of the TLC guidelines), except 2 servings red meat were replaced with different types of legumes 3 d/wk Control: legume-free TLC diet, macronutrient composition of 50–60% carbohydrates, 15% protein, and 25–35% of energy from fat	8 wk	Adiponectin concentration Leptin concentration	↑ Adiponectin concentration No significant change in leptin concentration
Saraf-Bank et al., 2015 [40]	Randomized crossover	Iran	26 men and women (age ≥ 30 y), first-degree relatives of patients with T2DM	Pinto beans and brown lentils	1-day dietary record	Intervention: habitual diet enriched with legumes, 4 packs (260 g)/wk; raw packs of pinto beans and brown lentils (65 g raw in 1 pack that is equivalent to 1 serving cooked) Control: habitual diet	6 wk	Inflammatory markers hs-CRP IL-6 TNF-α Serum levels of adiponectin FBS	↓ hs-CRP in all participants ↓ hs-CRP in female participants No significant change in IL-6, TNF-α, adiponectin, FBS
Saraf-Bank et al., 2016 [41]	Randomized crossover	Iran	26 men and women (mean age 50 ± 6.58 y), first-degree relatives of patients with T2DM	Pinto beans and brown lentils	1-day dietary record	Intervention: habitual diet enriched with legumes, 4 packs (260 g)/wk; raw packs of pinto beans and brown lentils (65 g raw in 1 pack that is equivalent to 1 serving cooked) Control: habitual diet	6 wk	Anthropometric measurements Glycemic indices HbA1c TAG TC HDL-C LDL-C SBP DBP	No significant change in anthropometric measurements, glycemic indices, blood pressure, and lipid profiles
Winham et al., 2007 [42]	Randomized crossover	US	16 men and women (age 22–65 y) with fasting insulin ≥ 15 uIU/mL	Pinto beans, black-eyed peas	24 h food recall	Group 1: 0.5 cup canned pinto beans/d Group 2: 0.5 cup canned black-eyed peas/d Control: 0.5 cup canned carrots/d Participants received test foods, recipes, and instructions not to eat other legumes, soy products, or carrots during the trial	8 wk	Serum lipoproteins Serum TC LDL-C HDL-C TG hs-CRP Glucose Insulin HbA1c	Pinto beans: ↓ TC ↓ LDL-C No significant change in TG, HDL, hs-CRP, glucose, insulin, and HbA1c
Zhang et al., 2011 [43]	Randomized crossover	US	64 men (age 35–75 y), nonsmoking with no history of IBD, stroke, diabetes, or colorectal or any cancers	Pinto, navy, kidney, lima, and black beans	24 h food recall	Intervention: legume-enriched diet, ~1.5 cups cooked beans/2000 kcal Control: isocaloric healthy US diet, calorie intake adjusted by study dietitians	4 wk	Plasma leptin Plasma ghrelin	↓ Plasma leptin No significant change in plasma ghrelin
Hosseinpour-Niazi et al., 2015 [44]	Randomized crossover	Iran	40 men and women (age 50–75 y) with T2DM, nonsmoking	Lentils, chickpeas, peas, beans	3-day dietary record	Intervention: non-soybean legume-based TLC diet, same as control diet except replacing 2 servings red meat with 1 cup cooked legumes (e.g., lentils, chickpeas, peas, and beans) 3 d/wk Control: isoenergetic legume-free TLC diet that followed the dietary recommendations of the TLC guidelines. This diet contained a macronutrient composition of 50–60% of energy from carbohydrates, 15% of energy from protein and 25–35% of energy from fat (<7% saturated fat, ≤20% MUFA, and ≤10% PUFA), as well as intake of 200 mg cholesterol and 25–30 g fiber	8 wk	Inflammatory markers hs-CRP IL-6 TNF-α	↓ hs-CRP ↓ IL-6 ↓ TNF-α
Hosseinpour-Niazi et al., 2015 [45]	Randomized crossover	Iran	32 men and women (age 58.1 ± 6.0 y) with T2DM, nonsmoking	Lentils, chickpeas, peas, beans	3-day dietary record	Intervention: non-soybean legume-based TLC diet, same as the control diet, except 2 servings of red meat were replaced with 1 cup cooked legumes (e.g., lentils, chickpeas, peas, and beans) for 3 d/wk Control: isoenergetic legume-free TLC diet that followed the dietary recommendations of the TLC guidelines. This diet contained a macronutrient composition of 50–60% of energy from carbohydrates, 15% of energy from protein and 25–35% of energy from fat (<7% saturated fat, ≤20% MUFA, and ≤10% PUFA), as well as intake of 200 mg cholesterol and 25–30 g fiber	8 wk	Cardiometabolic risk factors SBP DBP FBG Fasting insulin TG HDL-C LDL-C TC	↓ FBG ↓ Fasting insulin ↓ TG concentrations ↓ LDL-C
Hosseinpour-Niazi et al., 2022 [46]	Randomized crossover	Iran	300 men and women (mean age 55.4 y; mean BMI 30.4) with T2DM	Lentils, chickpeas, peas, beans	3-day dietary record	Intervention: legume-based DASH diet, similar to DASH diet, except 1 serving of bread was eliminated and 1 serving of red meat was replaced with 1 serving of legumes at least 5 d/wk Control: DASH diet (2000–3000 kcal based on energy requirement) that consisted of the following (servings/d): 8–12 fruits and vegetables, 7–15 whole grains, 2–3 low-fat dairy products, 2 red meat, 1 serving nuts and seeds, and limited intake of sweets and sugar (5 servings/wk)	16 wk	SBP DBP LDL-C HDL-C TC TG	↓ SBP No significant change in DBP, LDL-C, HDL-C, TC, TG
Alizadeh et al., 2014 [47]	Randomized parallel	Iran	42 premenopausal women	Beans (white, red, and wax), chickpeas, cowpeas, lentils, and split peas	Food diaries	Intervention: hypocaloric diet with 1 cup/d of cooked non-soy legumes, including beans (white, red, and wax), chickpeas, cowpeas, lentils, and split peas instead of meat Control: hypocaloric diet without legumes	6 wk	WC SBP DBP HOMA-IR ALT AST Fasting serum concentrations of TG LDL-C HDL-C FBS	↓ WC ↓ SBP ↓ ALT ↓ AST ↑ HOMA-IR ↑ Fasting concentration of insulin
Kazemi et al., 2020 [48]	Randomized parallel	Canada	55 women (age 18–35 y) with PCOS	Lentils, beans, split peas, and chickpeas	Face-to-face counseling	Intervention: pulse-based diet, including 2 standard pulse means daily containing 90 g split peas or 225 g chickpeas or beans or 150 g lentils Control: TLC diets that follow the dietary recommendations of the TLC guidelines	16 wk	Validated PCOS-specific HRQoL survey Metabolic and hormonal profile Weight	↑ HRQoL
Mollard et al., 2012 [49]	Randomized parallel	Canada	40 men and women (aged 35–55 y; BMI 28–39.9), nonsmoking	Lentils (Nupak), chickpeas (Nupak), yellow split peas (Nupak), and navy beans (Frema)	24 h food recall pulse log	Intervention: pulse diet; participants were provided 5 cups/wk (896 g) of prepared lentils (Nupak), chickpeas (Nupak), yellow split peas (Nupak), and navy beans (Ferma) Control: energy-restricted diet, adjusted based on participant 24 h food recall. Dietitians provided dietary guidance to achieve recommended macronutrient ranges (45%–65% energy from carbohydrates, 20%–35% energy from fat, 10%–35% energy from protein) and reduce energy intake by 2093 kJ/d (500 kcal/d) through decreasing fat, sugar, and alcohol intake and increasing fruit and vegetable intake and by portion control	8 wk	MetSyn risk factors WC SBP DBP HbA1c Postprandial C-peptide Fasting plasma leptin HDL LDL Fasting C-peptide TC TAG CRP Adiponectin GLP-1 and ghrelin	↓ WC ↓ SBP ↓ HbA1c ↑ HDL ↑ C-peptide No significant change in body weight, BMI, DBP, TC, LDL, TAG, CRP, adiponectin, GLP-1, or ghrelin
Safaeiyan et al., 2015 [50]	Randomized parallel	Iran	34 premenopausal women (age 20–50 y) with abdominal obesity and WC > 88 cm	Non-soy legumes, including red, white, and wax beans; cowpea, chickpeas, split peas; and lentil	3-day dietary record	Intervention: hypocaloric diet enriched with legumes, with 1 cup/d (2 servings/d) cooked non-soy legumes including beans (white, red, and wax), chickpeas, cowpeas, lentils, and split peas instead of meat Control: hypocaloric diet without legumes	6 wk	Cardiovascular risk factors TC LDL-C hs-CRP TAC NO Nitrite/nitrate MDA	↓ hs-CRP ↑ TAC (in first 3 wk)
Wilson et al., 2022 [51]	Randomized parallel	US	30 men and women (age 18–70 y) with abdominal obesity (WC ≥ 40 in. and ≥35 in., respectively) but without diabetes	Lentils	DHQ III 24 h food recall	Control: meals with meat and 0 g green lentils/wk Moderate-intake group: mixture of meat and lentils, including 300 g green lentils/wk High-intake group: strictly lentils as the protein source, including 600 g green lentils/wk Participants were asked to consume the same 5 provided meals each week but were otherwise asked to maintain their normal dietary and physical activity patterns	8 wk	HEI scores TC LDL HDL TG Impaired glucose tolerance (HOMA-IR, peripheral IR)	↓ HOMA-IR No significant change in peripheral IR, TC, LDL, HDL, or TG
Aslani et al., 2015 [52]	Randomized parallel	Iran	39 men and women (age 30–65 y) with overweight or obesity and T2DM	Lentil sprout	Weekly calls to confirm lentil sprout consumption	Intervention: received 60 g lentil sprout/d for 8 wk Control: continued regular diet	8 wk	Lipid profile TC LDL-C HDL-C TG ox-LDL	↓TG ↓ ox-LDL ↑ HDL-C

Abbreviations: ALT, alanine aminotransferase; AST, aspartate aminotransferase; BMI, body mass index (calculated as weight in kilograms divided by height in meters squared); CAT, catalase; CHD, coronary heart disease; CRP, C-reactive protein; CVD, cardiovascular disease; DASH, Dietary Approaches to Stop Hypertension; DBP, diastolic blood pressure; DHQ, diet history questionnaire; FBS, fasting blood sugar; FFQ, food frequency questionnaire; FRS, Framingham risk score; GLP, glucagon-like peptide; HbA1c, hemoglobin A1c; HDL-C, high-density lipoprotein cholesterol; HEI, Healthy Eating Index; HOMA-IR, homeostasis model of insulin resistance; HRQoL, health-related quality of life; hs-CRP, high-sensitivity C-reactive protein; IBD, inflammatory bowel disease; IL, interleukin; IR, insulin resistance; LDL-C, low-density lipoprotein cholesterol; MDA, malondialdehyde; MetSyn, metabolic syndrome; MI, myocardial infarction; MUFA, monounsaturated fatty acid; NHANES, US National Health and Nutrition Examination Survey; NO, nitric oxide; NA, not applicable; ox-LDL, oxidized low-density lipoprotein; PAD, peripheral artery disease; PCOS, polycystic ovary syndrome; PUFA, polyunsaturated fatty acid; Q1–Q4, quartiles 1–4; SBP, systolic blood pressure; sTNFR, soluble tumor necrosis factor receptor; TAC, total antioxidant capacity; T1–T3, tertiles 1–3; T2DM, type 2 diabetes mellitus; TAG, triacylglycerol; TC, total cholesterol; TLC, Therapeutic Lifestyle Changes; TNF, tumor necrosis factor; WC, waist circumference. Symbols indicate the following: ↑, increase; ↓, decrease. Mean values are presented ± SD.

**Table 3:** Matrix of pulse types reported in the included studies.

Reference	Study Design	Population	Pulse Intake									
			Mixture vs. Type ^a^	Pulse Type								
Askari et al., 2021 [23]	Cross-sectional	Men	Mixture	Lentil	Chickpea	Dry pea	Bean					Cotyledon
Baldeón et al., 2021 [24]	Cross-sectional	Men and women	Mixture	Lentil	Chickpea	Dry pea	Bean					Fava bean, lupin
Papanikolaou et al., 2008 [25]	Cross-sectional	Men and women	Mixture				Bean	Pinto bean	Kidney bean			
Pearce et al., 2021 [26]	Federated meta-analysis	Men and women	Mixture	Lentil	Chickpea	Dry pea	Bean					
Wennberg et al., 2015 [34]	Cohort	Men and women	Mixture	Lentil	Chickpea	Dry pea	Beans					
Liu et al., 2021 [29]	Cohort	Men and women	Mixture	Lentil	Chickpea	Dry pea, black-eyed pea	Beans			Black bean		
Miller et al., 2017 [30]	Cohort	Men and women	Mixture	Lentil	Chickpea	Dry pea, black-eyed pea	Beans			Black bean		
Nouri et al., 2016 [31]	Cohort	Men and women	Mixture	Lentil	Chickpea	Dry pea, black-eyed pea	Beans			Black bean		
Nouri et al., 2021 [32]	Cohort	Men and women	Mixture	Lentil		Dry pea	Beans					Mung bean
Guo et al., 2020 [28]	Cohort	Men and women	Mixture					Pinto bean	Red kidney bean			Mung, adzuki bean
Becerra-Tomás et al., 2018 [27]	Cohort	Men and women with high CVD risk	Type	Lentil	Chickpea		Dry bean					
Papandreou et al., 2019 [33]	Cohort	Men and women with high CVD risk	Type	Lentil	Chickpea		Dry bean					
Zahradka et al., 2013 [35]	Before-and-after	Men and women with PAD	Mixture	Lentil	Chickpea	Dry pea	Beans	Pinto bean	Kidney bean	Black bean	Navy bean	
Saraf-Bank et al., 2016 [41]	Randomized crossover	Men and women with high T2DM risk	Mixture	Lentil				Pinto bean				
Saraf-Bank et al., 2015 [40]	Randomized crossover	Men and women with high T2DM risk	Mixture	Lentil				Pinto bean				
Hosseinpour-Niazi et al., 2015 [44]	Randomized crossover	Men and women with T2DM	Mixture	Lentil	Chickpea	Dry pea	Beans					
Hosseinpour-Niazi et al., 2015 [45]	Randomized crossover	Men and women with T2DM	Mixture	Lentil	Chickpea	Dry pea	Beans					
Hosseinpour-Niazi et al., 2022 [46]	Randomized crossover	Men and women with T2DM	Mixture	Lentil	Chickpea	Dry pea	Beans					
Mirmiran et al., 2018 [38]	Randomized crossover	Men and women with T2DM	Mixture	Lentil	Chickpea	Dry pea	Beans					
Mirmiran et al., 2019 [39]	Randomized crossover	Men and women with T2DM	Mixture	Lentil	Chickpea	Dry pea	Beans					
Abeysekara et al., 2012 [36]	Randomized crossover	Men and women	Mixture	Lentil	Chickpea	Split pea		Pinto bean	Kidney bean	Black bean		Fava, broad bean
Hartman et al., 2010 [37]	Randomized crossover	Men	Mixture					Pinto bean	Kidney bean	Black bean	Navy bean	
Zhang et al., 2011 [43]	Randomized crossover	Men	Mixture					Pinto bean	Kidney bean	Black bean	Navy bean	Lima bean
Winham et al., 2007 [42]	Randomized crossover	Men and women with IR	Type			Black-eyed pea		Pinto bean				
Alizadeh et al., 2014 [47]	RCT	Women	Mixture	Lentil	Chickpea	Split pea	Beans (white, red, and wax)					Cowpea
Mollard et al., 2012 [49]	RCT	Men and women	Mixture	Lentil	Chickpea	Split pea					Navy bean	
Safaeiyan et al., 2015 [50]	RCT	Women with abdominal obesity	Mixture	Lentil	Chickpea	Split pea	Beans (white, red, and wax)					Cowpea
Kazemi et al., 2020 [48]	RCT	Women with PCOS	Mixture	Lentil	Chickpea	Split pea	Beans					
Wilson et al., 2022 [51]	RCT	Men and women with abdominal obesity	Type	Lentil								
Aslani et al., 2015 [52]	RCT	Men and women with T2DM	Type	Lentil sprout								

Abbreviations: CVD, cardiovascular disease; IR, insulin resistance; PAD, peripheral artery disease; PCOS, polycystic ovary syndrome; RCT, randomized controlled trial; T2DM, type 2 diabetes mellitus. ^a^ Mixture of pulses (non-soybean dry legumes) vs. isolated pulse type.

**Table 4:** Matrix of health outcomes reported in included studies.

Reference	Study Design	Population	Study Outcomes ^a^																
			Lipid Profile					Blood Pressure		CVD Risk and Mortality		Diabetes	Glycemic Control	MetSyn	Inflammatory and Oxidative Stress Biomarkers			Hormonal Profile	Other
Askari et al., 2021 [23]	Cross-sectional	Men	[↓] LDL-C	[↑] HDL-C		[—] TAG				[—] FRS			[—] FBS						
Baldeón et al., 2021 [24]	Cross-sectional	Men and women	[—] LDL-C	[—] HDL-C	[—] TC		[—] TG	[↓] SBP	[↓] DBP			[↓] T2DM		[↓] Incident MetSyn [—] WC					
Papanikolaou et al., 2008 [25]	Cross-sectional	Men and women						[↓] SBP						[↓] WC					
Guo et al., 2020 [28]	Cohort	Men and women						[—] Hypertension											
Pearce et al., 2021 [26]	Federated meta-analysis	Men and women										[—] Incident T2DM							
Becerra-Tomás et al., 2018 [27]	Cohort	Men and women with high CVD risk										[↓] Incident T2DM							
Wennberg et al., 2015 [34]	Cohort	Men and women										[↓] Incident T2DM in women	[↓] Impaired glucose tolerance or impaired fasting glucose in women	[↑] WC in men					
Liu et al., 2021 [29]	Cohort	Men and women								[↓] Incident CVD [—] Incident cancer	[↓] All-cause mortality [↓] Cancer mortality [—] CVD mortality								
Miller et al., 2017 [30]	Cohort	Men and women								[—] Incident CVD	[↓] All-cause mortality [↓] Non-CVD mortality [—] CVD mortality								
Nouri et al., 2016 [31]	Cohort	Men and women								[↓] Incident CVD events									
Nouri et al., 2021 [32]	Cohort	Men and women								[↓] Incident CVD events									
Papandreou et al., 2019 [33]	Cohort	Men and women with high CVD risk									[↓] Cancer mortality [↓] CVD mortality								
Zahradka et al., 2013 [35]	Before-and-after	Men and women with PAD	[—] LDL-C		[—] TC [↓] Serum cholesterol					[↓] Ankle–brachial index			[—] FBS						
Saraf-Bank et al., 2016 [41]	Randomized crossover	Men and women with high T2DM risk	[—] LDL-C	[—] HDL-C	[—] TC	[—] TAG		[—] SBP	[—] DBP				[—] HbA1c						
Winham et al., 2007 [42]	Randomized crossover	Men and women with IR	[↓] LDL-C	[—] HDL-C	[↓] TC		[—] TG						[—] HbA1c			[—] hs-CRP			
Abeysekara et al., 2012 [36]	Randomized crossover	Men and women	[↓] LDL-C	[—] HDL-C	[↓] TC	[—] TAG										[—] CRP			
Hartman et al., 2010 [37]	Randomized crossover	Men											[↑] Fasting glucose			[↓] CRP		[—] sTNFRI [—] sTNFRII [—] C-peptide	
Saraf-Bank et al., 2015 [40]	Randomized crossover	Men and women with high T2DM risk											[—] FBS			[↓] hs-CRP	[—] IL-6 [—] TNF-α [—] Adiponectin		
Hosseinpour-Niazi et al., 2015 (a) [44]	Randomized crossover	Men and women with T2DM														[↓] hs-CRP	[↓] IL-6 [↓] TNF)-α		
Hosseinpour-Niazi et al., 2015 (b) [45]	Randomized crossover	Men and women with T2DM	[↓] LDL-C		[—] TC		[↓] TG	[—] SBP	[—] DBP				[↓] Fasting glucose [↓] Fasting insulin						
Hosseinpour-Niazi et al., 2022 [46]	Randomized crossover	Men and women with T2DM	[—] LDL-C	[—] HDL-C	[—] TC		[—] TG	[↓] SBP	[—] DBP										
Alizadeh et al., 2014 [47]	RCT	Women	[—] LDL-C	[—] HDL-C			[—] TG	[↓] SBP	[—] DBP				[—] FBS [↑] HOMA-IR	[↓] WC					[↓] ALT [↓] AST
Mollard et al., 2012 [49]	RCT	Men and women	[—] LDL-C	[↑] HDL-C	[—] TC	[—] TAG		[↓] SBP	[—] DBP				[↓] HbA1c	[↓] WC			[—] Adiponectin	[↑] C-peptide [—] Leptin [—] Ghrelin [—] GLP-1	
Safaeiyan et al., 2015 [50]	RCT	Women with abdominal obesity	[—] LDL-C		[—] TC										[—] NO [—] MDA	[↓] hs-CRP		[↑] TAC [—] Nitrite/nitrate	
Wilson et al., 2022 [51]	RCT	Men and women with abdominal obesity	[—] LDL	[—] HDL	[—] TC		[—] TG						[↓] HOMA-IR [—] Peripheral IR						
Aslani et al., 2015 [52]	RCT	Men and women with T2DM	[—] LDL-C	[↑] HDL-C	[—] TC		[↓] TG								[↓] ox-LDL				
Kazemi et al., 2020 [48]	RCT	Women with PCOS																	[↑] HRQoL
Mirmiran et al., 2018 [38]	Randomized crossover	Men and women with T2DM													[↓] Serum MDA [↓] Serum ox-LDL [↑] Serum NO [↑] CAT activity				
Mirmiran et al., 2019 [39]	Randomized crossover	Men and women with T2DM															[↑] Adiponectin	[—] Leptin	
Zhang et al., 2011 [43]	Randomized crossover	Men																[↓] Leptin [—] Ghrelin	

Abbreviations: ALT, alanine aminotransferase; AST, aspartate aminotransferase; CAT, catalase; CRP, C-reactive protein; CVD, cardiovascular disease; DBP, diastolic blood pressure; FBS, fasting blood sugar; FRS, Framingham risk score; GLP, glucagon-like peptide; HbA1c, hemoglobin A1c; HDL-C, high-density lipoprotein cholesterol; HOMA-IR, homeostasis model of insulin resistance; HRQoL, health-related quality of life; hs-CRP, high-sensitivity C-reactive protein; IL, interleukin; IR, insulin resistance; LDL-C, low-density lipoprotein cholesterol; MDA, malondialdehyde; MetSyn, metabolic syndrome; NO, nitric oxide; ox-LDL, oxidized low-density lipoprotein; PAD, peripheral artery disease; PCOS, polycystic ovary syndrome; RCT, randomized controlled trial; SBP, systolic blood pressure; sTNFR, soluble tumor necrosis factor receptor; T2DM, type 2 diabetes mellitus; TAC, total antioxidant capacity; TAG, triacylglycerol; TC, total cholesterol; TNF, tumor necrosis factor; WC, waist circumference. ^a^ Symbols indicate the following: ↑, increase; ↓, decrease; —, no change. Shading indicates similar study measures.

## Data Availability

The data presented in this study are available on request from the corresponding author due to privacy reasons.

## Supplementary Materials

The following supporting information can be downloaded at: https://www.mdpi.com/article/10.3390/nu16101435/s1, File S1: Search History.
