# Supplementary material for: Pulse Consumption and Health Outcomes: A Scoping Review
Source: Nutrients. 2024 May 9;16(10):1435. doi: 10.3390/nu16101435 (PMC11124391; doi:10.3390/nu16101435)
Supplement: Supplementary file 1 [file nutrients-16-01435-s001.zip › nutrients-2985033-supplementary.pdf]

Supplemental Material File S1. Search History

Database Summary

Database	Platform	Years covered	Date conducted	# results
Medline	PubMed	1946 - 2023	7/21/2023	3724
Cochrane Library Cochrane Database of Systematic Reviews, Cochrane Central Register of Controlled Trials	Wiley	Issue 7 of 12, July 2023	7/21/2023	1214
EMBASE	Elsevier	1974-2023	7/21/2023	3299
Web of Science SCI-EXPANDED, SSCI, AHCI, CPCI-S, CPCI-SSH, BKCI-S, BKCI-SSH, ESCI	Clarivate Analytics	1900-2023	7/21/2023	5603
Total				13840
With duplicates removed				8697

PubMed

#3	#1 AND #2	3724
#2	"Health Status"[Majr] OR "Cardiovascular Diseases"[Majr] OR "Diabetes Mellitus"[Majr] OR "Body Weight"[Majr] OR Health Outcome*[ti] OR Health Status[ti] OR Health Effect*[ti] OR Cardiovascular[ti] OR Cardiac[ti] OR Heart Disease*[ti] OR Blood Pressure[ti] OR Diabetes[ti] OR Blood Glucose[ti] OR Insulin Resistance[ti] OR Obesity[ti] OR Body Mass Index[ti] OR Bmi[ti] OR Weight[ti]	3440688
#1	(("Vigna"[Mesh] OR "Fabaceae"[Mesh:NoExp]) AND ("Diet"[MeSH] OR "Eating"[Mesh])) OR (Pulse*[tiab] OR Legume*[tiab] OR Bean*[tiab] OR Lentil*[tiab] OR Dry pea*[tiab] OR Chickpea*[tiab] OR Cow pea*[tiab] OR Cowpea*[tiab] OR Black eyed pea*[tiab] OR Blackeyed pea*[tiab] OR Black gram*[tiab] OR Masha[tiab] OR Vigna radiata[tiab] OR Vigna aconitifolia[tiab] OR Vigna angularis[tiab] OR Vigna unguiculata[tiab] OR Vigna mungo[tiab] OR Phaseolus mungo[tiab] OR Vigna umbellata[tiab]) AND (intake[tiab] OR Consumption[tiab] OR Diet[tiab] OR Ingestion[tiab] OR Eat[tiab] OR Eating[tiab])	17159

History and Search Details Download Delete

Search	Actions	Details	Query	Results	Time
#3	•••	>	Search: **#1 AND #2** Sort by: **Most Recent**	3,724	15:04:35
#2	•••	>	Search: **"Health Status"[Majr] OR "Cardiovascular Diseases"[Majr] OR "Diabetes Mellitus"[Majr] OR "Body Weight"[Majr] OR Health Outcome*[ti] OR Health Status[ti] OR Health Effect*[ti] OR Cardiovascular[ti] OR Cardiac[ti] OR Heart Disease*[ti] OR Blood Pressure[ti] OR Diabetes[ti] OR Blood Glucose[ti] OR Insulin Resistance[ti] OR Obesity[ti] OR Body Mass Index[ti] OR Bmi[ti] OR Weight[ti]** Sort by: **Most Recent**	3,440,688	15:03:47
#1	•••	>	Search: **(("Vigna"[Mesh] OR "Fabaceae"[Mesh:NoExp]) AND ("Diet"[MeSH] OR "Eating"[Mesh])) OR (Pulse*[tiab] OR Legume*[tiab] OR Bean*[tiab] OR Lentil*[tiab] OR Dry pea*[tiab] OR Chickpea*[tiab] OR Cow pea*[tiab] OR Cowpea*[tiab] OR Black eyed pea*[tiab] OR Blackeyed pea*[tiab] OR Black gram*[tiab] OR Masha[tiab] OR Vigna radiata[tiab] OR Vigna aconitifolia[tiab] OR Vigna angularis[tiab] OR Vigna unguiculata[tiab] OR Vigna mungo[tiab] OR Phaseolus mungo[tiab] OR Vigna umbellata[tiab]) AND (intake[tiab] OR Consumption[tiab] OR Diet[tiab] OR Ingestion[tiab] OR Eat[tiab] OR Eating[tiab])** Sort by: **Most Recent**	17,159	15:03:38

Showing 1 to 3 of 3 entries

Embase

#5	#3 and #4	3299
#4	'health status'/mj OR 'cardiovascular disease'/mj OR 'diabetes mellitus'/mj OR 'body weight'/mj OR 'health outcome*':ti OR 'health status':ti OR 'health effect*':ti OR cardiovascular:ti OR cardiac:ti OR 'heart disease*':ti OR 'blood pressure':ti OR diabetes:ti OR 'blood glucose':ti OR 'insulin resistance':ti OR obesity:ti OR 'body mass index':ti OR bmi:ti OR weight:ti	1662691
#3	#1 OR #2	23661
#2	(pulse*:ti,ab,kw OR legume*:ti,ab,kw OR bean*:ti,ab,kw OR lentil*:ti,ab,kw OR 'dry pea*':ti,ab,kw OR chickpea*:ti,ab,kw OR 'cow pea*':ti,ab,kw OR cowpea*:ti,ab,kw OR 'black eyed pea*':ti,ab,kw OR 'blackeyed pea*':ti,ab,kw OR 'black gram*':ti,ab,kw OR masha:ti,ab,kw OR 'vigna radiata':ti,ab,kw OR 'vigna aconitifolia':ti,ab,kw OR 'vigna angularis':ti,ab,kw OR 'vigna unguiculata':ti,ab,kw OR 'vigna mungo':ti,ab,kw OR 'phaseolus mungo':ti,ab,kw OR 'vigna umbellata':ti,ab,kw) AND (intake:ti,ab,kw OR consumption:ti,ab,kw OR diet:ti,ab,kw OR ingestion:ti,ab,kw OR eat:ti,ab,kw OR eating:ti,ab,kw)	23438
#1	('vigna'/exp OR 'fabaceae'/de) AND ('eating'/exp OR 'diet'/exp)	493

History Save | Delete | Print view | Export | Email Combine > using And Or

#5	#3 AND #4
#4	**'health status'**/mj OR **'cardiovascular disease'**/mj OR **'diabetes mellitus'**/mj OR **'body weight'**/mj OR **'health outcome*'**:ti OR **'health status'**:ti OR **'health effect*'**:ti OR **cardiovascular**:ti OR **cardiac**:ti OR **'heart disease*'**:ti OR **'blood pressure'**:ti OR **diabetes**:ti OR **'blood glucose'**:ti OR **'insulin resistance'**:ti OR **obesity**:ti OR **'body mass index'**:t [illegible] **weight**:ti
#3	#1 OR #2
#2	(**pulse***:ti,ab,kw OR **legume***:ti,ab,kw OR **bean***:ti,ab,kw OR **lentil***:ti,ab,kw OR **'dry pea*'**:ti,ab,kw OR **chickpea***:ti,ab,kw OR **'cow pea*'**:ti,ab,kw OR **cowpea***:ti,ab,kw OR **'black eyed pea*'**:ti,ab,kw OR **'blackeyed pea*'**:ti,ab,kw OR **'black gram*'**:ti,ab,kw OR **masha**:ti,ab,kw OR **'vigna radiata'**:ti,ab,kw OR **'vigna aconitifolia'**:ti,ab,kw OR **'vigna angular** [illegible] **'vigna unguiculata'**:ti,ab,kw OR **'vigna mungo'**:ti,ab,kw OR **'phaseolus mungo'**:ti,ab,kw OR **'vigna umbellata'**:ti,ab,kw) AND (**intake**:ti,ab,kw OR **consumption**:ti,ab,kw OR **diet**:ti,ab,kw OR **ingestion**:ti,ab,kw OR **eat**:ti,ab,kw OR **eating**:ti,ab,kw)
#1	(**'vigna'**/exp OR **'fabaceae'**/de) AND (**'eating'**/exp OR **'diet'**/exp)

Web of Science

#1	((Pulse* OR Legume* OR Bean* OR Lentil* OR "Dry pea*" OR Chickpea* OR "Cow pea*" OR Cowpea* OR "Black eyed pea*" OR "Blackeyed pea*" OR "Black gram*" OR Masha OR "Vigna radiata" OR "Vigna aconitifolia" OR "Vigna angularis" OR "Vigna unguiculata" OR "Vigna mungo" OR "Phaseolus mungo" OR "Vigna umbellata") AND (intake OR Consumption OR Diet OR Ingestion OR Eat OR Eating)) (Topic) and "Health Outcome*" OR "Health Status" OR "Health Effect*" OR Cardiovascular OR Cardiac OR "Heart Disease*" OR "Blood Pressure" OR Diabetes OR "Blood Glucose" OR "Insulin Resistance" OR Obesity OR "Body Mass Index" OR Bmi OR Weight (Title)	5603

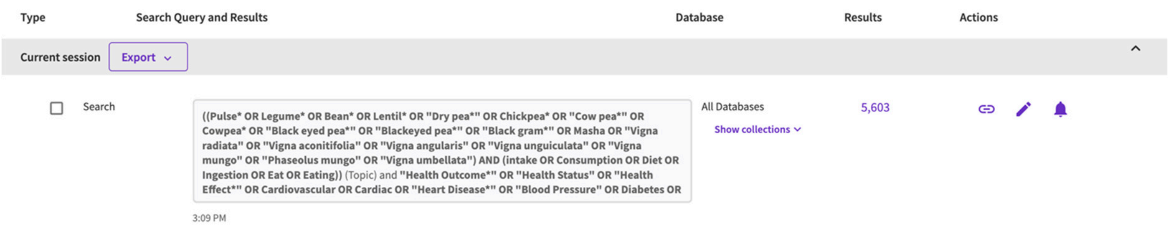

Cochrane

#1	(Pulse* OR Legume* OR Bean* OR Lentil* OR Dry NEXT pea* OR Chickpea* OR Cow NEXT pea* OR Cowpea* OR Black NEXT eyed NEXT pea* OR Blackeyed NEXT pea* OR Black NEXT gram* OR Masha OR "Vigna radiata" OR "Vigna aconitifolia" OR "Vigna angularis" OR "Vigna unguiculata" OR "Vigna mungo" OR "Phaseolus mungo" OR "Vigna umbellata") AND (intake OR Consumption OR Diet OR Ingestion OR Eat OR Eating) in Title Abstract Keyword AND Health NEXT Outcome* OR "Health Status" OR Health NEXT Effect* OR Cardiovascular OR Cardiac OR Heart NEXT Disease* OR "Blood Pressure" OR Diabetes OR "Blood Glucose" OR "Insulin Resistance" OR Obesity OR "Body Mass Index" OR Bmi OR Weight in Record Title	1214

Cochrane Reviews	Cochrane Protocols	Trials	Editorials	Special Collections	Clinical Answers	More
16	0	1198	0	0	0	

16 Cochrane Reviews matching **(Pulse* OR Legume* OR Bean* OR Lentil* OR Dry NEXT pea* OR Chickpea* OR Cow NEXT pea* OR Cowpea* OR Black NEXT eyed NEXT pea* OR Blackeyed NEXT pea* OR Black NEXT gram* OR Masha OR "Vigna radiata" OR "Vigna aconitifolia" OR "Vigna angularis" OR "Vigna unguiculata" OR "Vigna mungo" OR "Phaseolus mungo" OR "Vigna umbellata") AND (intake OR Consumption OR Diet OR Ingestion OR Eat OR Eating) in Title Abstract Keyword AND Health NEXT Outcome* OR "Health Status" OR Health NEXT Effect* OR Cardiovascular OR Cardiac OR Heart NEXT Disease* OR "Blood Pressure" OR Diabetes OR "Blood Glucose" OR "Insulin Resistance" OR Obesity OR "Body Mass Index" OR Bmi OR Weight in Record Title - (Word variations have been searched)**

Cochrane Database of Systematic Reviews
Issue 7 of 12, July 2023
